# Supplementary material for: Observation of ballistic upstream modes at fractional quantum Hall edges of graphene
Source: Nat Commun. 2022 Jan 11;13:213. doi: 10.1038/s41467-021-27805-4 (PMC8752686; doi:10.1038/s41467-021-27805-4)
Supplement: Supplementary file 1 — Supplementary Information [file 41467_2021_27805_MOESM1_ESM.pdf]

Supplementary Information for "Observation of ballistic upstream modes at fractional quantum Hall edges of graphene"

Ravi Kumar[1], Saurabh Kumar Srivastav[1], Christian Spånslätt[2,3,4], K. Watanabe[5], T. Taniguchi[5], Yuval Gefen[6], Alexander D. Mirlin[3,4,7,8], and Anindya Das[1]

[1] *Department of Physics, Indian Institute of Science, Bangalore 560012, India*
[2] *Department of Microtechnology and Nanoscience, Chalmers University of Technology, S-41296 Göteborg, Sweden*
[3] *Institut for Quantum Materials and Technology, Karlsruhe Institute of Technology, 76021 Karlsruhe, Germany*
[4] *Institut für Theorie der Kondensierten Materie, Karlsruhe Institute of Technology, 76128 Karlsruhe, Germany*
[5] *National Institute of Material Science, 1-1 Namiki, Tsukuba 305-0044, Japan*
[6] *Department of Condensed Matter Physics, Weizmann Institute of Science, Rehovot 76100, Israel*
[7] *Petersburg Nuclear Physics Institute, 188300 St. Petersburg, Russia and*
[8] *L. D. Landau Institute for Theoretical Physics RAS, 119334 Moscow, Russia*

PACS numbers:

This Supplementary Information contains the following details:

S1. Device Fabrication and characteristics
S2. Edge structure of $\nu = 3/5$ FQH state.
S3. Quantum Hall characterization
S4. Noise measurement setup
S5. Noise data for shorter length
S6. Gain and electron temperature estimation
S7. Noise analysis
S8. Robustness of upstream noise across the $\nu = 2/3$ plateau
S9. Energy gap of FQH states.
S10. Device-2 data for $\nu = 2/3$ and $\nu = 3/5$ states
S11. Electrical conductance for no charge equilibration using Landauer-Büttiker model
S12. Bias response of $\nu = 1/3$ state
S13. Theoretical calculation of noise for $\nu = 2/3$ and $\nu = 3/5$ states

S1. DEVICE FABRICATION AND CHARACTERISTICS

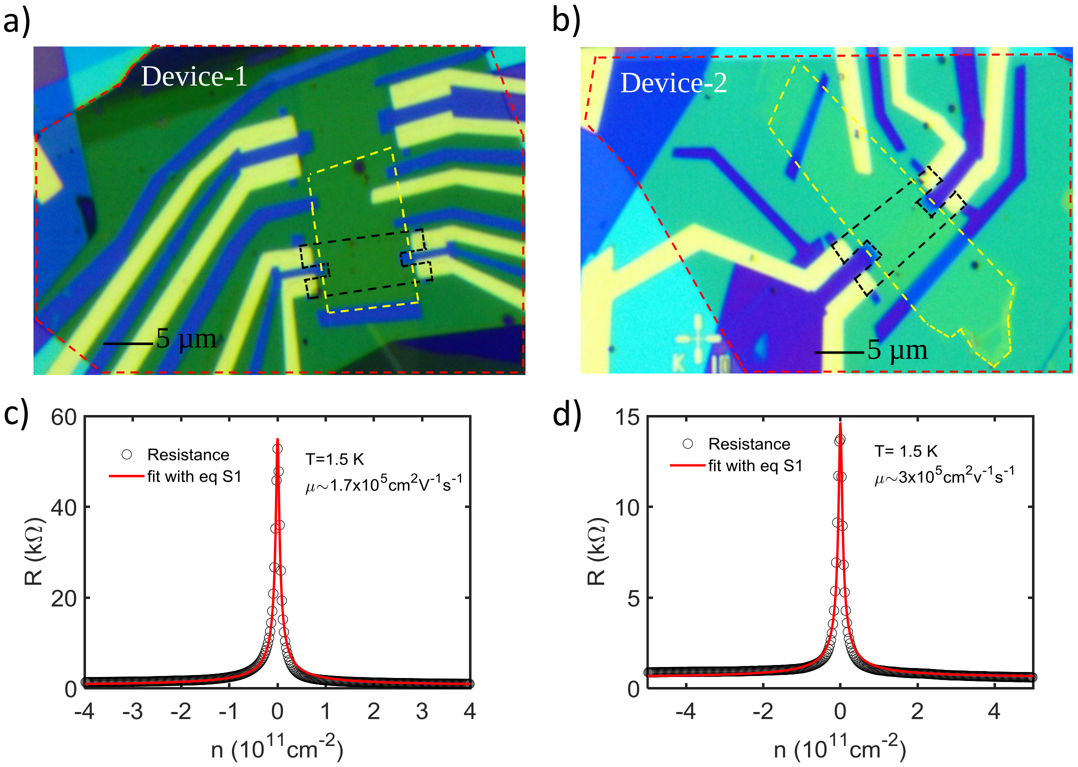

FIG. S1: **Device image and characteristics.** (**a**) and (**b**) show optical images of devices 1 and 2, respectively. Region of BLG, top graphite, and bottom graphite are marked by black, yellow, and red dashed lines, respectively. (**c**) and (**d**) show back-gate response of two-probe resistance of device 1 and device 2, respectively, at zero magnetic field and 1.5K. Solid circles show the experimental data and the red curve is the fit of data in accordance to Eq. (S1). The fit gives mobility of $\sim 1.7 \times 10^5 \mathrm{cm}^2\mathrm{V}^{-1}\mathrm{s}^{-1}$ and $\sim 3 \times 10^5 \mathrm{cm}^2\mathrm{V}^{-1}\mathrm{s}^{-1}$ for device 1 and device 2, respectively.

For making encapsulated devices (heterostructure of graphite/hBN/bilayer graphene(BLG)/hBN/graphite), we used standard dry transfer pick-up technique [S1, S2]. Fabrication of these heterostructure involved mechanical exfoliation of hBN and graphite crystals on oxidized silicon wafer using the widely used scotch-tape technique. BLG and graphite flakes were exfoliated from natural graphite crystals. Suitable flakes were identified under the optical microscope. First, a graphite of thickness ~ 5 nm which works as top gate or screening layer, is picked up at 90°C using a Poly-Bisphenol-A-Carbonate (PC) coated Polydimethylsiloxane (PDMS) stamp placed on a glass slide, attached to tip of a home-built micromanipulator. Subsequently, this graphite flake is aligned on top of an already exfoliated hBN flake of thickness ~ 20 nm at 90°C, and due to better adhesion of hBN with graphite than SiO_2, hBN flake gets picked up. Next, we align this graphite/hBN stamp on top of a BLG flake. BLG was picked up at the same 90°C temperature. Next step involved the pick-up of bottom hBN (~ 20 nm). Bottom hBN was picked up using the previously picked-up graphite/hBN/BLG following the previous process. This graphite/hBN/BLG/hBN heterostructure was used to pick up the bottom graphite flake following previous step. Finally, this resulting heterostructure (graphite/hBN/BLG/hBN/graphite) was dropped down on top of a silicon wafer with 285 nm thick SiO_2 on top,at temperature 180°C. To remove the residues of PC, the final stack was cleaned in chloroform ($CHCl_3$) overnight followed by cleaning in acetone and and iso-propyl alcohol (IPA). The BLG channel area of the stack was microscopically ironed using an AFM (atomic force microscopy) tip in contact mode [S3], to remove any atomic level strain or ripples or small bubbles from the channel area, which can arise due to stacking process. After this, Poly-methyl-methacrylate (PMMA) photoresist was coated on this heterostructure to define the contacts using electron beam lithography (EBL). After EBL, reactive ion etching (mixture of CHF_3 and O_2 gas with flow rate of 40 sccm and 4 sccm, respectively, at 25°C with RF power of 60W) was used to define the edge contact. The etching time was optimized such that the bottom hBN does not get etched out completely to isolate the contacts from bottom graphite flake, which was used as the back gate. Then, thermal deposition of Cr/Pd/Au (4/12/60 nm) was done in a evaporator chamber having base pressure of $\sim 1 - 2 \times 10^{-7}$ mbar. After deposition, lift-off procedure was performed in hot acetone and IPA. Finally,

to define the BLG edge along the shorter length, we again performed reactive ion etching. The optical images of the devices are shown in Fig.S1(a) and Fig.S1(b), respectively. The two-terminal resistances (R) of device 1 and 2 were measured as a function of back-gate voltage at zero magnetic field and plotted as a function of charge carrier density (n), as shown in Fig. S1(c) and Fig. S1(d), respectively. Here, n is given by $\frac{C_{BG}(V_{BG}-V_{CN})}{e}$ with C_{BG} and V_{CN} being the capacitance per unit area of bottom graphite gate and the voltage at the charge-neutrality point, respectively. The measured data is fitted with the equation

$$R = R_C + \frac{L}{We\mu\sqrt{(n_0^2 + (\frac{C_{BG}(V_{BG}-V_{CN})}{e})^2)}}, \tag{S1}$$

where R_C, L, W, μ, e, and n_0 are the contact resistance, length, width, mobility, electron charge, and charge inhomogeneity, respectively. The resistance data is fitted with Eq. (S1) and the extracted mobility for device 1 and device 2 is found to be $\sim 1.7 \times 10^5 \mathrm{cm}^2\mathrm{V}^{-1}\mathrm{s}^{-1}$ and $\sim 3 \times 10^5 \mathrm{cm}^2\mathrm{V}^{-1}\mathrm{s}^{-1}$, respectively. The high mobility of the device is necessary to observe fractional states.

S2. EDGE STRUCTURE OF $\nu = 3/5$ FQH STATE

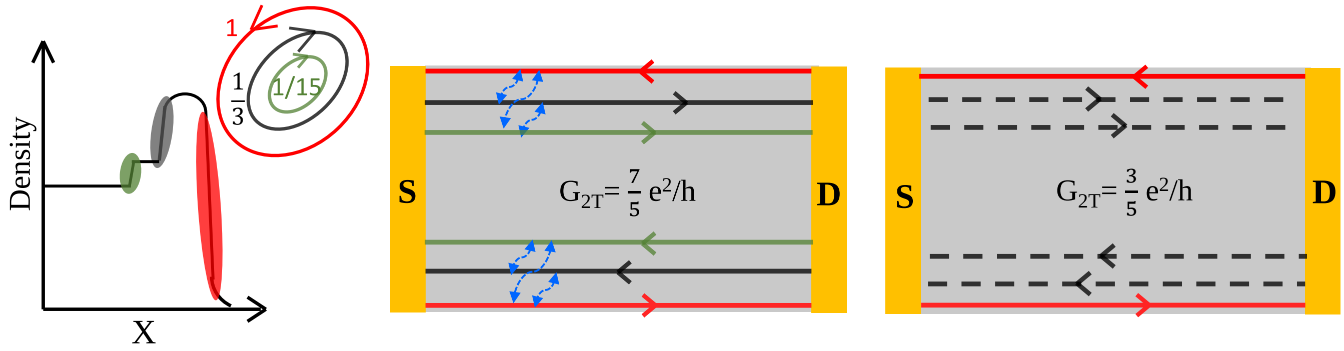

FIG. S2: **Edge structure for $\nu = 3/5$ FQH state**. Left panel shows the density profile at the edge of a sample with counter-propagating edge structure for $\nu = 3/5$. X is the radial coordinate of the droplet. Downstream mode is the red line, while upstream modes are the black and green lines. The conductance of red, black, and green modes are $1e^2/h$, $1/3e^2/h$, and $1/15e^2/h$, respectively. Middle panel visualizes the regime of sample length $L < l_{\mathrm{eq}}^C$, where l_{eq}^C is the charge equilibration length, with the two-terminal conductance of $G_{2T} = (7/5)e^2/h$ for 3/5 state. Right panel shows schematically the regime $l_{\mathrm{eq}}^C < L < l_{\mathrm{eq}}^H$, with the two-terminal conductance of $G_{2T} = (3/5)e^2/h$. In this regime, the charge flows only downstream (red line, conductance $(3/5)e^2/h$), while the upstream transport is neutral, i.e., carries only heat (dashed black lines).

S3. QUANTUM HALL CHARACTERIZATION

We characterize QH response of the device-1 for both the chiralities of QH edge and for different edge length of the device as used in the noise measurements. Details are shown in below Fig. S3.

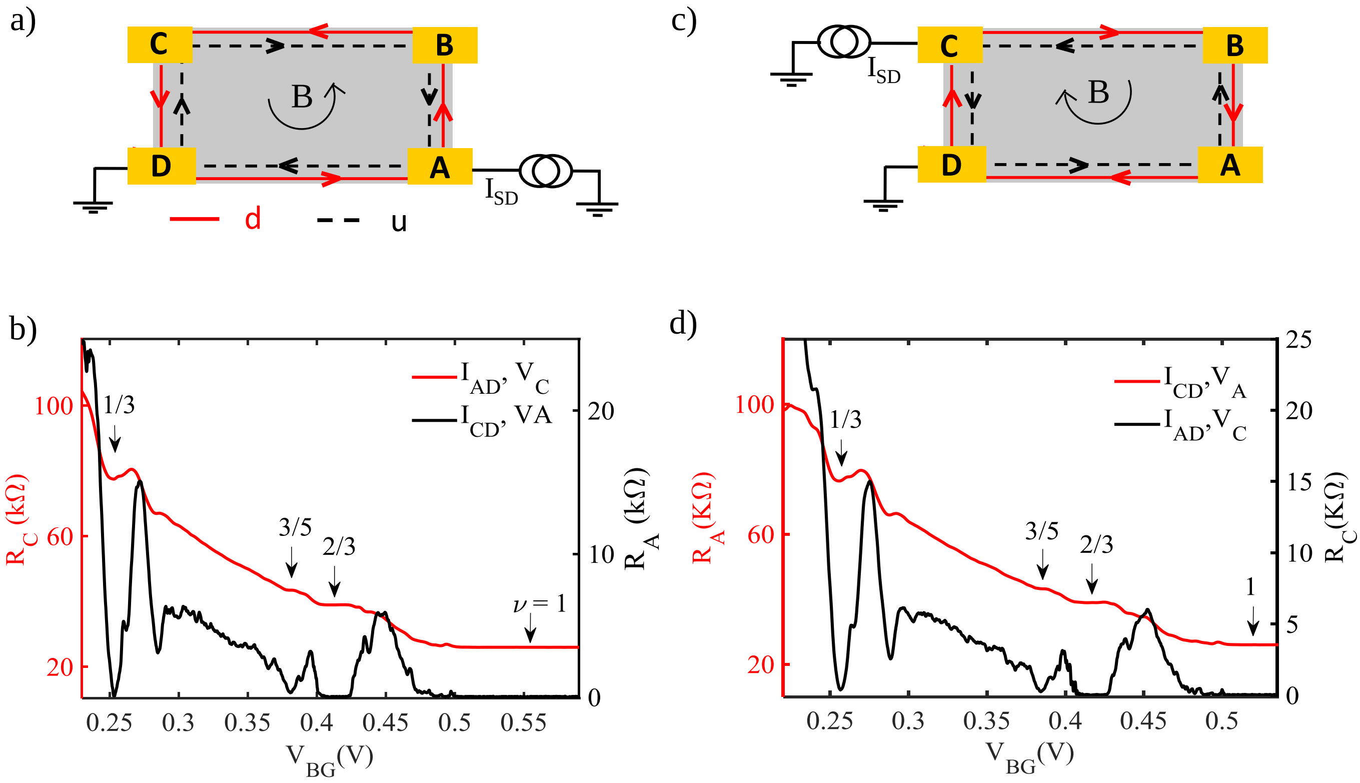

FIG. S3: (**a**) Measurement scheme for checking low-frequency QH response for anticlockwise chirality (+10 T). An ac current $I_{\rm SD}$ of 2 nA magnitude at frequency f = 13 Hz from Lock-In is injected at contact A, contact D is cold ground, and voltage is measured at contact C. The measured voltage is converted to resistance, shown as R_C (red color) in panel (b). Similarly, 2 nA ac current at 13 Hz is injected at contact C and resistance is measured at contact A, shown as R_A (black color) in panel (b). We see clear quantized plateaus in R_C and vanishing resistance in R_A at $\nu = 1$, 2/3, and R_A reaches $\sim 30-40\Omega$ for 1/3. (**c**) Measurement scheme for checking low frequency QH response for clockwise chirality(−10 T). An ac current $I_{\rm SD}$ of 2 nA magnitude at frequency f = 13 Hz from Lock-In is injected at contact C, contact D is cold ground, and resistance is measured at contact A, shown as R_A (red color) in panel (d). Similarly, 2 nA ac current at 13 Hz is injected at contact A and resistance is measured at contact C, shown as R_C (black color) in panel (d). We see clear quantized plateaus in R_A and vanishing resistance in R_C at $\nu = 1$ and 2/3.

S4. NOISE MEASUREMENT SETUP

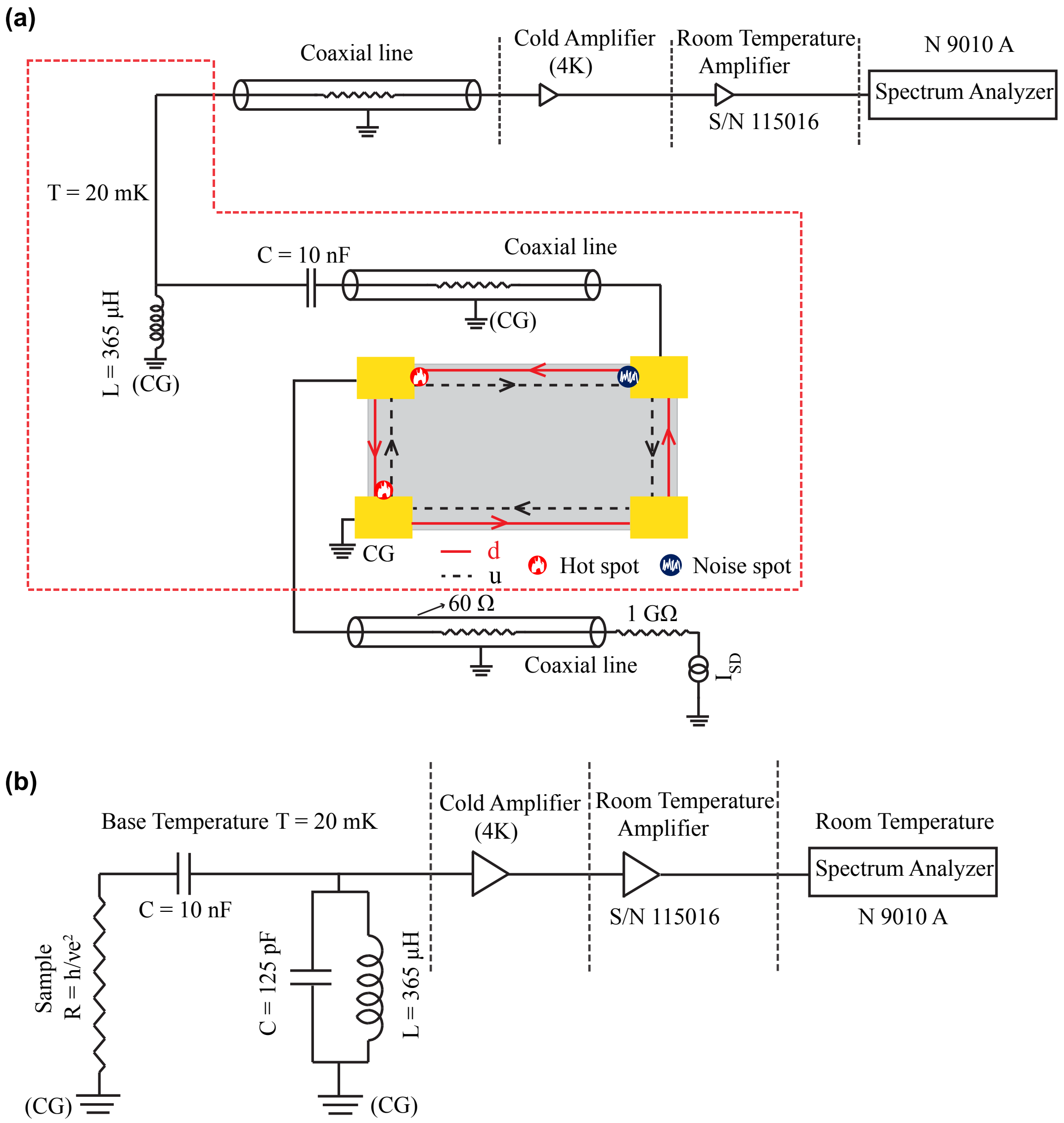

FIG. S4: **Experimental setup for noise measurement.** (**a**) Schematic of the measurement setup. The device was mounted on a chip carrier which was connected to the cold finger fixed to the mixing chamber plate of dilution refrigerator. The ground contact pins are directly shorted to the cold finger to achieve the cold ground. The sample was current biased with a 1 GΩ resistor located at the top of dilution fridge. Current fluctuations measured at contact located along the upstream direction are converted on chip into voltage fluctuations using the quantum Hall (QH) resistance $R = h/\nu e^2$ [as shown schematically in figure (**b**)] where ν is the filling factor. The voltage noise generated from the device is filtered using a superconducting resonant LC tank circuit with resonance frequency of 763kHz and bandwidth 30kHz. The filtered signal is then amplified by a home made cryogenic voltage pre-amplifier which was thermalized to 4K plate of dilution refrigerator. This pre-amplified signal was then amplified using a voltage amplifier (PR-E3-SMA S/N 115016) placed at the top of the fridge at room temperature. After second stage of amplification, amplified signal was measured using a spectrum analyzer (N9010A). All the noise measurements were done using the band width of $\sim$ 30 kHz. The resonant LC tank circuit was built using inductor L of $\sim$ 365 μH made from a superconducting coil thermally anchored to the mixing chamber of dilution refrigerator. The parallel C of $\sim$ 125 pF is the capacitance that develops along the coaxial lines connecting the sample to the cryogenic pre-amplifier. A ceramic capacitance of 10 nF was introduced between sample and inductor to block the DC current along the measurement line. The typical input voltage noise and current noise of cryogenic pre-amplifiers were $\sim$ 250 pV/$\sqrt{\text{Hz}}$ and $\sim$ 20-25 fA/$\sqrt{\text{Hz}}$, respectively. (**b**) The schematic circuit diagram of the measurement setup.

S5. NOISE DATA FOR SHORTER LENGTH

To perform noise measurements along the shorter length of the device-1, we reversed the chirality of the QH edge by changing the magnetic field from $+10\,$T to $-10\,$T. Measurement scheme for this is shown in panel (**a**). Before noise measurements, we again check the linearity of the bias response of the $\nu = 2/3$ state as well as the downstream character of the charge propagation by injecting a 100 pA AC signal from Lock-In on top of the DC bias current at contact A, contact D is cold grounded, and voltage is measured at contacts A and B, as shown in panel (**e**), cf. Fig. 2c of the main manuscript. Flat V_A indicates that the conductance of $\nu = 2/3$ state is independent of bias current. Flat zero value of V_B shows that the charge propagates entirely downstream and only energy can propagate upstream. To detect the upstream heat transport, a noiseless dc current is injected at contact A, and noise is measured at contact B along the upstream direction. Due to downstream current I_A, a hot spot is created at the backside of the contact A. Part of this heat is carried by the upstream mode towards the noise spot near the contact B, thus generating noise at contact B. The measured noise for $\nu = 2/3$ and $\nu = 1$ is shown in the panel (**b**). For $\nu = 1$, no noise is detected, in full consistency with the absence of upstream modes at this filling. For $\nu = 2/3$, the noise is nearly identical to that for $L = 10\,\mu$m, see Fig. 2d of the main manuscript. In panel (**c**), an alternative configuration is shown: we inject noiseless dc current at contact C and measure noise at contact B. The contact A is electrically floating, thus cutting the path along the edge from the hot spot at D to the noise spot at B. No noise is detected for $\nu = 2/3$ state, see panel (**d**), confirming that the heat propagation responsible for the noise generation takes place along the edge, cf. Fig. 2e of the main manuscript.

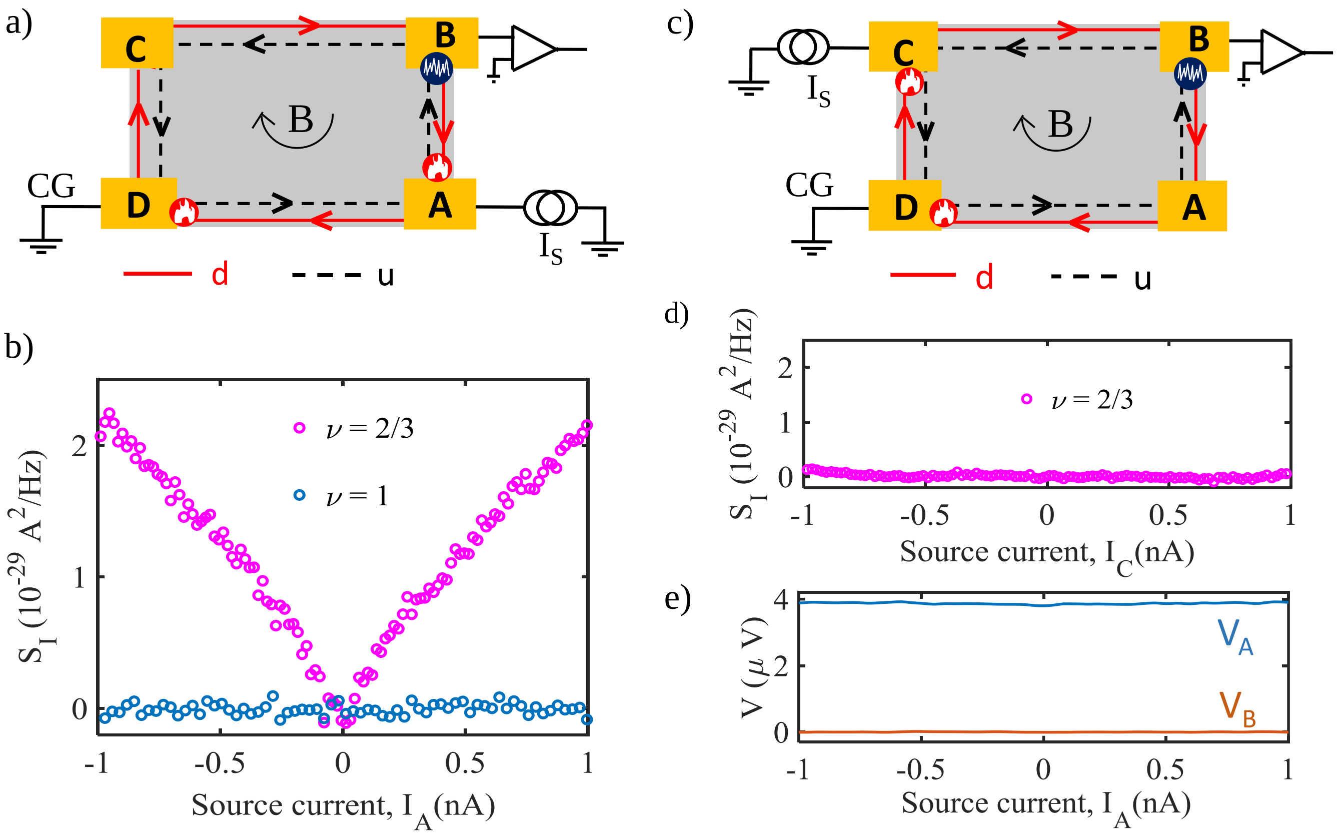

FIG. S5: (**a**) Device measurement scheme for upstream noise detection along the shorter length ($4\mu m$) of the device. Device is set into $\nu = 2/3$ state where d and u represent counter-propagating eigen-modes. (**b**)Upstream noise for hole-conjugate state $\nu = 2/3$ and electron-like state $\nu = 1$, measured along the shorter length. (**c**) and (**d**) represent measurement scheme and the measured noise at 2/3 for an alternate contacts configuration, respectively . (**e**) Bias response of 2/3 state.

S6. GAIN AND ELECTRON TEMPERATURE ESTIMATION

We have estimated the gain of amplification chain and the electron temperature from temperature-dependent Johnson-Nyquist noise (thermal noise). At zero impinging current, the equilibrium voltage noise spectrum is given by

$$S_V = g^2(4k_BTR + V_n^2 + i_n^2R^2)BW\,, \tag{S2}$$

where g is the total gain of amplification chain, k_B the Boltzmann constant, T the bath temperature (temperature of mixing chamber (MC) plate), R is the resistance of quantum hall state, V_n^2 and i_n^2 are the intrinsic voltage and current noise of the amplifier, and BW is the frequency bandwidth. The first term, $4k_BTR$ corresponds to the thermal noise. At a quantum Hall plateau, any change in bath temperature will only affect the first term in Eq. (S2), while all other terms are independent of temperature. If one plots the $\frac{S_V}{BW}$ as a function of temperature, the slope of the linear curve will be equal to $4g^2k_BR$. Since at quantum Hall plateau the resistance R is exactly known, one can easily extract the gain of the amplification chain from the slope and the intrinsic noise of the amplifier from the intercept. The gain is found using the following equation:

$$g = \sqrt{\Big(\frac{\partial\big(\frac{S_V}{BW}\big)}{\partial T}\Big)\Big(\frac{1}{4k_BR}\Big)}\,, \tag{S3}$$

where $\left(\frac{\partial\left(\frac{S_V}{BW}\right)}{\partial T}\right)$ is the slope of the linear fit. The implementation of this procedure is shown in Fig.S6 and Fig.S7 for $\nu = 1$ and $2/3$, respectively.

The noise spectrum (S_V) at zero impinging current measured on the $\nu = 1$ plateau at different bath temperatures is shown as a function of frequency in Fig. S6(a). The S_V value at the resonance frequency, divided by BW, is plotted as a function of bath temperature in Fig. S6(b), where the red solid line is the linear fit to the data in thew temperature range from 175mK to 1K. From the slope, we extract the gain, which is found to be ~ 1250. Note that we do not use the base temperature data for the fitting in Fig.S6b, because the electron temperature (T_e) could be different from the base temperature. As the gain is known, one can calculate the $(V_n^2 + i_n^2R^2)$ from the intercept of the linear fitting of S_v/BW vs temperature. Now from the known value of the measured noise at the base temperature, the corresponding electron temperature (T_e) can be found directly using the following equation:

$$T_e = \frac{\left(\left(\frac{S_V}{g^2BW}\right) - (V_n^2 + i_n^2R^2)\right)}{4k_BR}\,. \tag{S4}$$

The measured value of noise for $\nu = 1$ at base temperature is $1.1 \times 10^{-8}V^2$ in Fig. S6(a), which corresponds to $T_e = 27mK$, which is consistent with the electron temperature measured in our previous work [S10]. The fact that T_e is very close to the bath temperature can be also seen directly from Fig, S6(b): the $T_{bath} = 20mK$ data point (a star symbol) is located very close to the dashed line representing the extrapolation of the linear fit into the region below 175 mK.

We further perform the similar analyis for the $\nu = 2/3$ QH plateau. The voltage noise spectrum measured at $\nu = 2/3$ at resonance frequency along the upstream direction (for $L = 4\mu$m) at different bath temperatures is shown, as a function of bias current, in Fig. S7(a). We calculate S_V at zero bias (shown as red circle) by averaging 10 data points on each side of the zero bias current. We then divide averaged S_V at zero bias by the bandwidth and plot the result as a function of the bath temperature in Fig. S7(b). Following the same procedure as described above, we calculate the gain and the electron temperature T_e. This yields the gain ~ 1240 and the electron temperarure $T_e = 22\,$mK for the lowest bath temperature $T_{\mathrm{bath}} = 20\,$mK. It can be noticed that both the $\nu = 1$ and $\nu = 2/3$ give very similar results. The fact that the electro cooling on the $\nu = 2/3$ plateau remains fully efficient down to our lowest bath temperature is clearly manifested in Fig. S7b: the noise data points for T_{bath} equal to 20 mK, 40 mK, and 50 mK (star symbols) fall nearly perfectly on the linear extrapolation (dashed line) of the fit into the region below 60 mK. This confirms that T_{bath} and T_e are indeed very close in our setup.

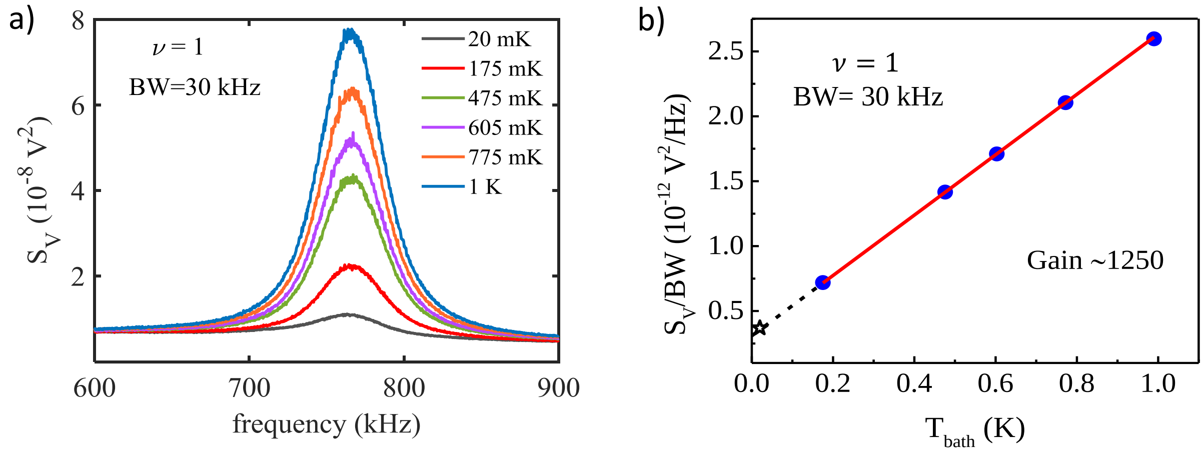

FIG. S6: **Gain and electron temperature estimation for $\nu = 1$ QH plateau.** (**a**) Noise S_V measured by spectrum analyzer is plotted as a function of frequency at different bath temperature for $\nu = 1$. From this plot, resonance frequency of tank circuit was found to be ∼763 kHz. (**b**) Symbols represent the noise S_V divided by bandwidth (BW) at resonance frequency as a function of bath temperature. Solid red line is the linear fit to the data from 175 mK to 1K and the dashed line is the linear extrapolation below 175 mK. Using Eq. (S3) and the slope of this linear fit, the gain g was found to be equal to ∼1250.

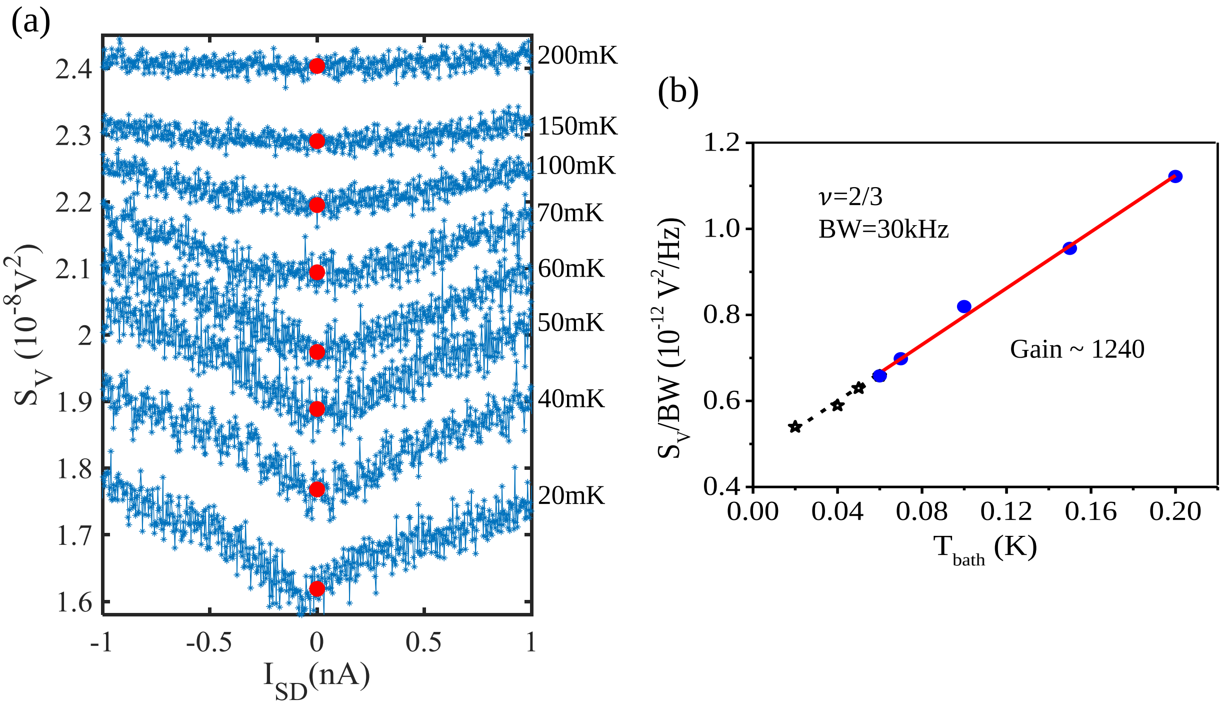

FIG. S7: **Gain and electron temperature estimation for $\nu = 2/3$ QH plateau.** (**a**) Noise S_V measured by spectrum analyzer at resonance frequency is plotted as a function of bias current at different bath temperatures at $\nu = 2/3$ QH plateau. The data sets corresponding to 100 mK, 150 mK, and 200 mK are divided by factor of 1.12, 1.25, and 1.4, respectively, for the ease of comparison in the same plot. The red circle in each data set represents the average value of 10 data points on each side of the zero bias current. (**b**) Symbols represent the averaged noise S_V at zero bias divided by bandwidth (BW) as a function of bath temperature. Solid red line is the linear fit to the data from 60 mK to 200 mK, and the dashed line is the linear extrapolation below 60 mK. Using Eq. (S3) and the slope of this linear fit, the gain g was found to be equal to ~1240.

S7. NOISE ANALYSIS

The excess noise (δS_V) due to bias current, measured along the upstream direction, is calculated by subtracting the noise value at zero bias current from the noise at finite bias, i.e $\delta S_V = S_V(I) - S_V(I = 0)$. The excess voltage noise is converted to excess current noise by dividing δS_V by the square of the resistance value at QH state ($S_I = \frac{\delta S_V}{R^2}$,where $R = \frac{h}{\nu e^2}$). In Fig. S8a and S8b, we show the excess upstream noise at $\nu = 2/3$ for $L = 4\mu$m and $L = 10\mu$m, for different bath temperatures. We fit the noise data linearly for both positive and negative bias current, and from the slopes we extract the value of the noise at $I_{\mathrm{SD}} = \pm 1nA$. The resulting values are shown in Fig. 3(c) of the manuscript. The linear fittings of the data at different temperatures are shown in Fig. S8c.

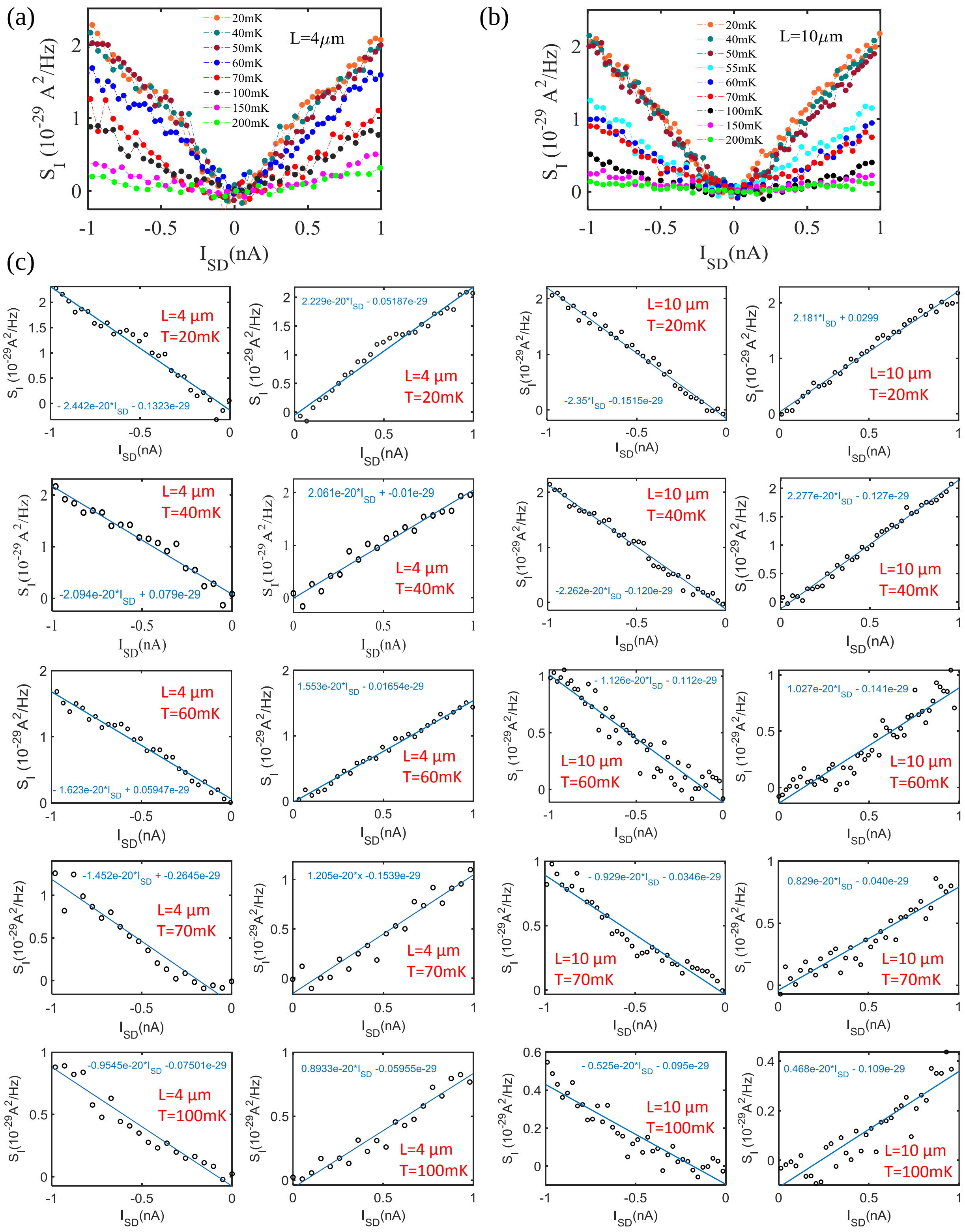

FIG. S8: **Noise analysis.** **(a)** and **(b)** show the noise measured in the upstream direction at $\nu = 2/3$ for $L = 4\mu$m and $L = 10\mu$m, respectively. **(c)** shows the linear fits of the noise data for both positive and negative bias at several temperatures.

S8. ROBUSTNESS OF NOISE ACROSS THE $\nu = 2/3$ PLATEAU

The noise at the $\nu = 2/3$ state is measured at different gate voltages inside the 2/3 plateau for both chiralities of the QH edge. The noise remains the same, as shown in Fig.S9.

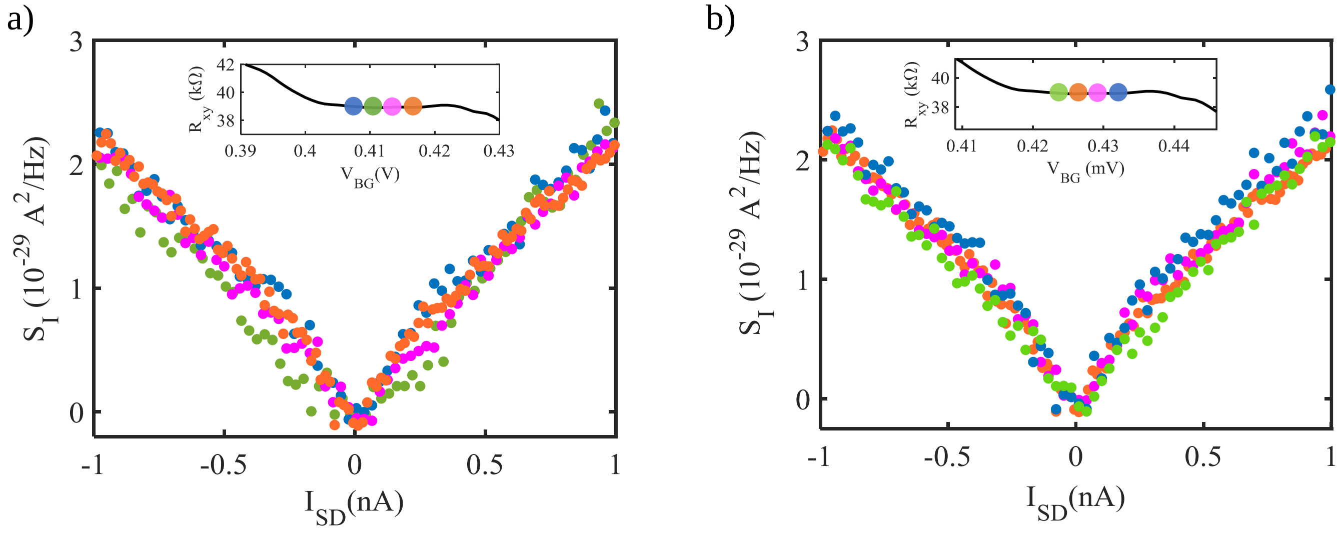

FIG. S9: (**a**) and (**b**) show the noise measured along the longer length (10 μm) and shorter length (4 μm) of the device, respectively, at different values of the density (controlled by the back gate voltage V_{BG}) on the $\nu = 2/3$ plateau. Insets show the Hall-resistance plateau of the $\nu = 2/3$ state. Different colors in noise data correspond to different values of the gate voltage inside the plateau, as marked by the corresponding color dots in the insets.

S9. ENERGY GAP OF FQH STATES

Here (Fig. S10) we present experimental determination of the energy gap of $\nu = 1/3$ and $\nu = 2/3$ states by temperature activated behavior of the longitudinal resistance R_{xx} at the fixed magnetic field 10 T.

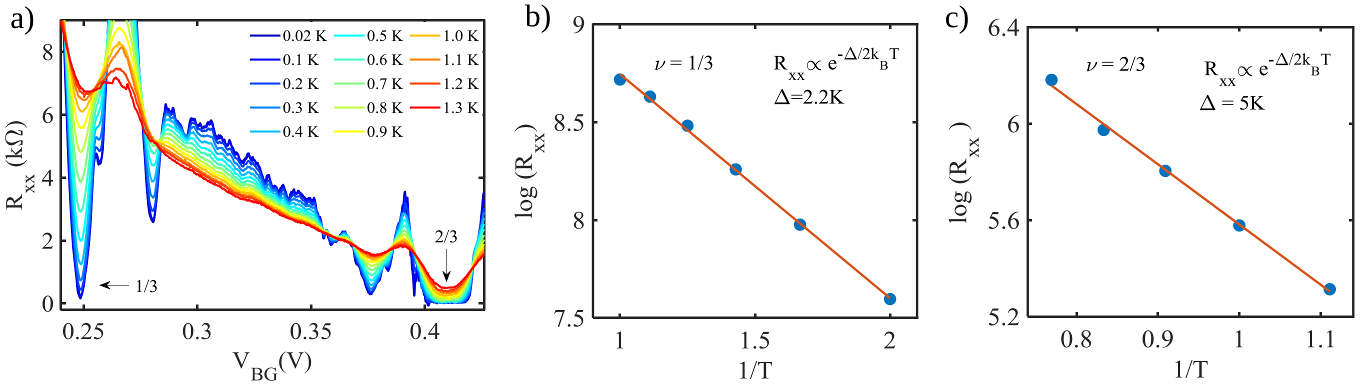

FIG. S10: **Activation energy gap of $\nu = 1/3$ and $\nu = 2/3$ state.** (**a**) Gate dependence of the longitudinal resistance R_{xx} measured at 10 T magnetic field at different temperatures, starting from base temperature of 20 mK up to 1.3 K. (**b**) Logarithm of R_{xx} vs inverse temperature $1/T$ for $\nu = 1/3$ state. The linear fit yields $R \propto \exp(-\Delta/2k_BT)$ with $\Delta = 2.2$ K. (**c**)Logarithm of R_{xx} vs inverse temperature $1/T$ for $\nu = 2/3$ state. The linear fit yields $R \propto \exp(-\Delta/2k_BT)$ with $\Delta = 5$ K.

S10. DEVICE-2 DATA

The noise measurements for the $\nu = 2/3$ state were also carried out in device-2 (shown in Fig. S1b) for 10 μm propagation length. Furthermore, we were able to measure in device-2 noise for the $\nu = 3/5$ state too, due to a better longitudinal response (more pronounced quantum Hall plateau) of 3/5 state in device-2. Data is shown in Fig. S11. The results for the $\nu = 2/3$ state (Fig. S11b) are in full agreement with those for device-1. The results for the $\nu = 3/5$ state (Fig. S11c) confirm the existence of upstream modes for this state as well. The magnitude of the noise for the $\nu = 3/5$ state is rather close to that for the $\nu = 2/3$ state and is in a very good agreement with the theoretical prediction.

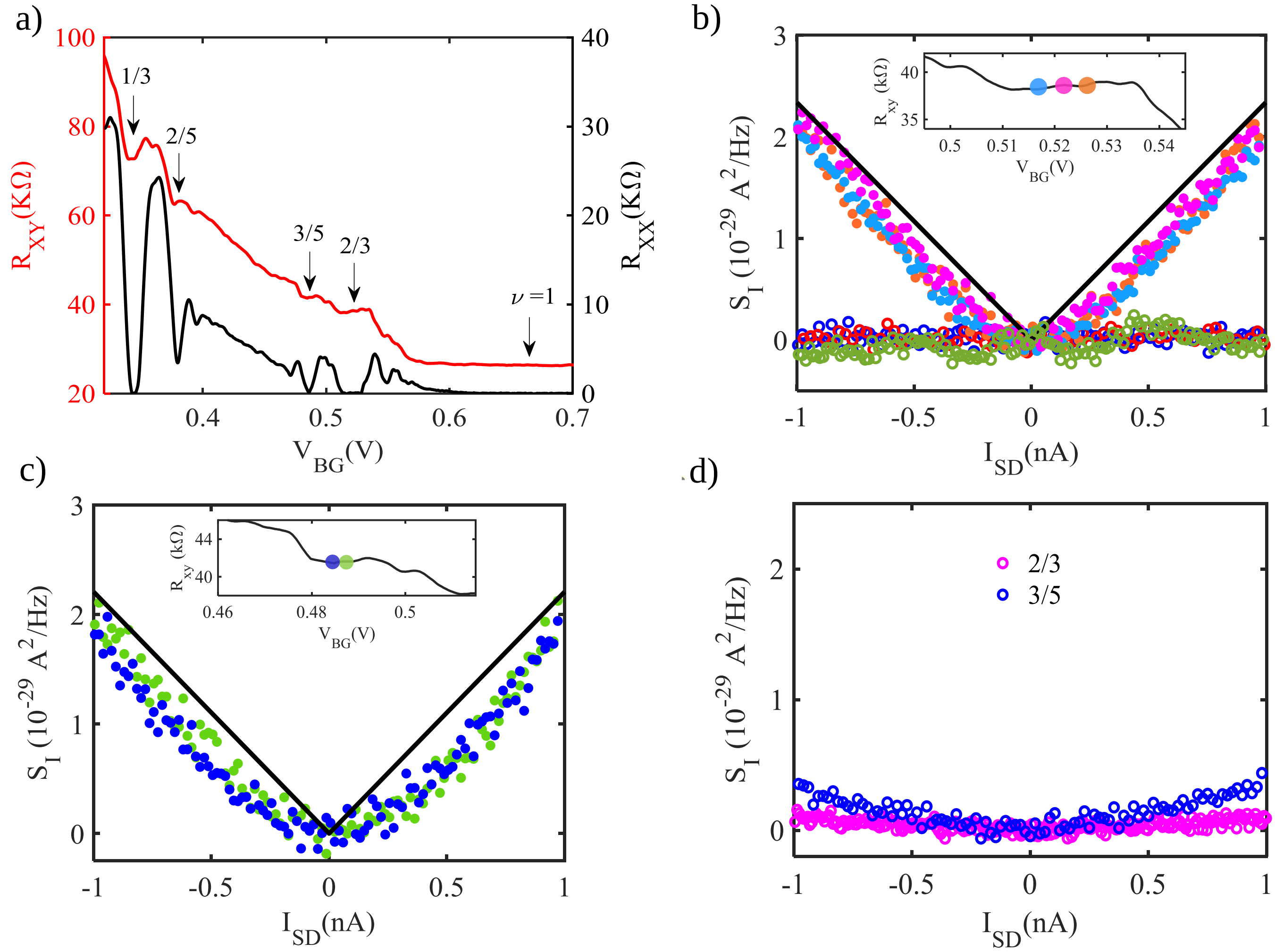

FIG. S11: **Device-2 data.** (**a**) Quantum Hall response of device-2. (**b**) Noise data for $\nu = 2/3$, 1, and 2. Solid circles correspond to noise for 2/3, with different color data corresponding to different gate voltage position inside the 2/3 plateau as shown in the inset. The value of the noise perfectly agrees with that for device-1, see Fig. 2d of the main manuscript, and with the theoretical prediction (solid black line) $S_I = 0.146\, I_{\rm SD}$, see Eq. (S43) below. Open olive, blue and red circles correspond to the noise for $\nu = 1/3$, 1 and 2, respectively. No noise is detected, in full agreement with the absence of upstream modes for these fillings. (**c**) Noise for $\nu = 3/5$. The magnitude of the noise agrees very well with the theoretical prediction (solid black line), $S_I = 0.138\, I_{\rm SD}$, see Eq. (S56) below. (**d**) Noise for $\nu = 2/3$ and 3/5 in alternative configuration of contacts, with the upstream path from hot spot to the noise spot cut by a floating metallic contact. For $\nu = 2/3$, no noise is detected, as in device-1, demonstrating that the heat propagation from hot spot to noise spot, which is responsible for the noise generation, takes place along the edge. For $\nu = 3/5$ a very weak noise is detected, which is apparently due to a contribution of heat transport through the bulk, possibly via the mechanism identified in Ref. [S4]. While this contribution is totally negligible for the $\nu = 2/3$ state, it becomes detectable for the $\nu = 3/5$ in view of a smaller value of the gap.

S11. ELECTRICAL CONDUCTANCE IN THE ABSENCE OF CHARGE EQUILIBRATION USING LANDAUER-BÜTTIKER FORMALISM

As demonstrated in the main text, the charge propagates only downstream in our devices, which implies that the charge equilibration length is much smaller than the relevant propagation length L ($4\,\mu$m or $10\,\mu$m). Here we provide further evidence of strong charge equilibration in our devices. For this purpose, we calculate resistances assuming no charge equilibration in two configuration of contacts. We show that these values are very different from experimentally observed resistances that, on the other hand, are in full agreement with the values predicted for a regime of full charge equilibration.

To calculate the value of electrical conductance of the $\nu = 2/3$ state without charge equilibration, we follow the approach of Landauer-Büttiker model [S5]. Note that in this calculation we assume full equilibration at the contacts. We calculate the electrical conductance of 2/3 state, which host counter propagating edge states of conductance $1e^2/h$ and $(1/3)e^2/h$. The schematic of the device with two different contact configurations is shown in Fig. S12a,b.

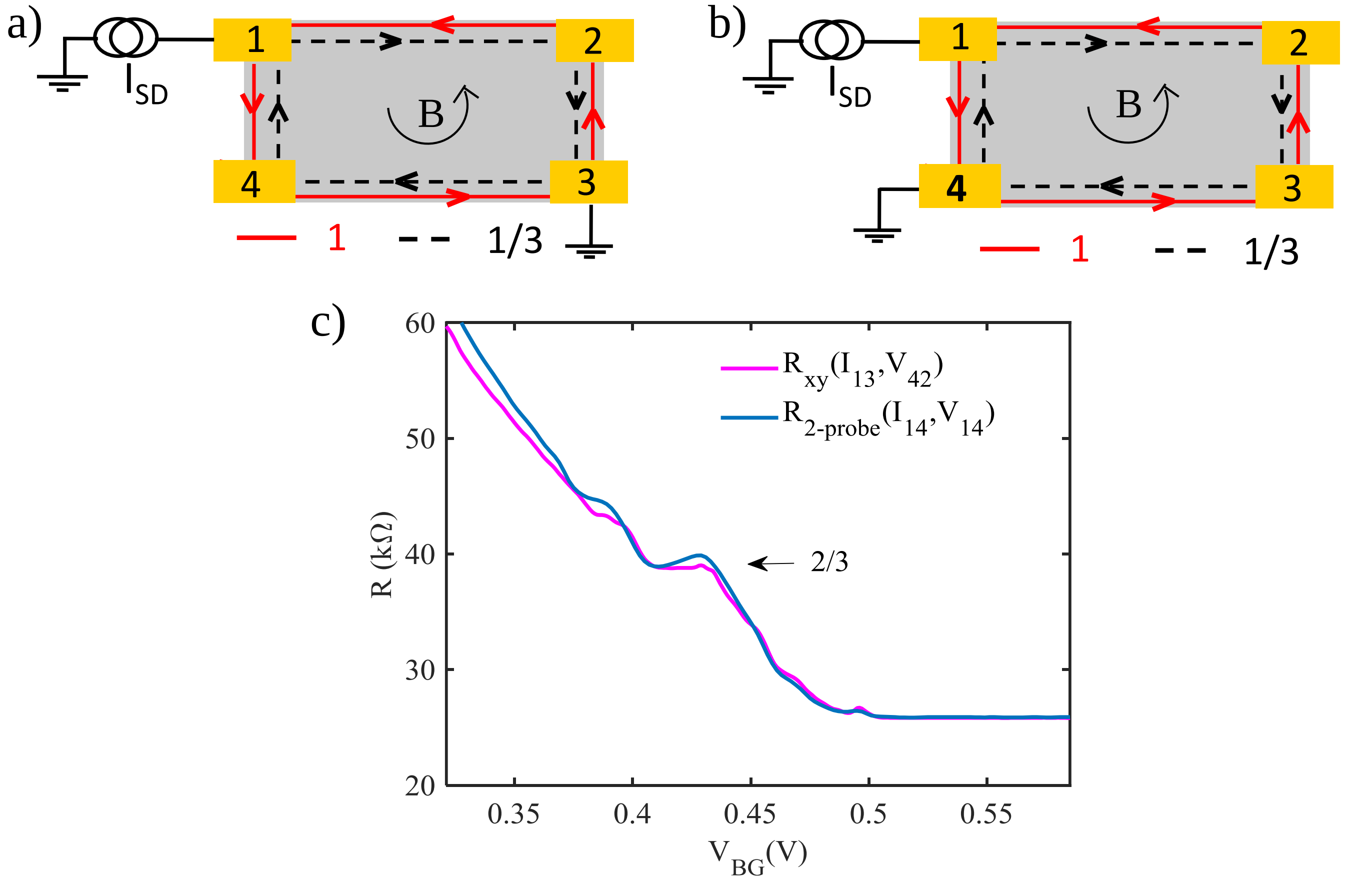

FIG. S12: (**a**) Contact configurations to measure transverse Hall resistance of 2/3 state. The contact 1 is biased, 3 is grounded, 2 and 4 floating. (**b**) An alternative contact configuration: 1 is biased, 4 is grounded, 2 and 3 floating. (**c**) Measured transverse Hall resistance R_{xy} (magenta color) for the first contact configuration and resistance at contact 1 (blue color) for the second contact configuration. Legend notations: the first index in the subscript of I corresponds to the current-fed contact and the second one to the ground contact. Indices in the subscript of V correspond to contacts across which the potential difference is measured.

For a multi-probe device, the net current flowing in i^{th} contact is given by

$$I_i = \sum_j (G_{j\leftarrow i}V_i - G_{i\leftarrow j}V_j)\,, \tag{S5}$$

where $G_{j\leftarrow i}$ is the conductance from i^{th} contact to j^{th} contact and V_i is the voltage of ith contact. In the absence of

charge equilibration along the edges, we would have

$$\begin{pmatrix} I_1 \\ I_2 \\ I_3 \\ I_4 \end{pmatrix} = \frac{e^2}{h} \begin{pmatrix} (1+1/3) & -1 & 0 & -1/3 \\ -1/3 & (1+1/3) & -1 & 0 \\ 0 & -1/3 & 1+1/3 & -1 \\ -1 & 0 & -1/3 & (1+1/3) \end{pmatrix} \begin{pmatrix} V_1 \\ V_2 \\ V_3 \\ V_4 \end{pmatrix} . \tag{S6}$$

Eliminating the rows and column associated with contact 3 from Eq. (S6), we get

$$\begin{pmatrix} I_1 \\ I_2 \\ I_4 \end{pmatrix} = \frac{e^2}{h} \begin{pmatrix} 4/3 & -1 & -1/3 \\ -1/3 & 4/3 & 0 \\ -1 & 0 & 4/3 \end{pmatrix} \begin{pmatrix} V_1 \\ V_2 \\ V_4 \end{pmatrix} . \tag{S7}$$

The current is injected at contact 1, so that

$$\begin{pmatrix} I_1 \\ I_2 \\ I_4 \end{pmatrix} = \begin{pmatrix} I \\ 0 \\ 0 \end{pmatrix} . \tag{S8}$$

According to Eq. (S7), this yields the voltages

$$\begin{pmatrix} V_1 \\ V_2 \\ V_4 \end{pmatrix} = I \frac{h}{e^2} \begin{pmatrix} 1.2 \\ 0.3 \\ 0.9 \end{pmatrix} , \tag{S9}$$

so that the voltages measured at contacts 1, 2,and 4 will be

$$V_1 = 1.2 \times I \frac{h}{e^2} , \qquad V_2 = 0.3 \times I \frac{h}{e^2} , \qquad V_4 = 0.9 \times I \frac{h}{e^2} . \tag{S10}$$

Hence the transverse Hall resistance in the absence of charge equilibration is given by

$$R_{xy} = \frac{V_4 - V_2}{I} = 0.6 \times \frac{h}{e^2} = 15.5\,\mathrm{k}\Omega . \tag{S11}$$

At the same time, the experimentally measured value of R_{xy} is $\approx 38.6k\Omega$, as shown in Fig. S9(c) in magenta color.

Next we consider the configuration shown in Fig. S9(b). Here the contact 4 is grounded. After eliminating the rows and column associated with contact 4 from the matrix Eq. (S6), we get

$$\begin{pmatrix} I_1 \\ I_2 \\ I_3 \end{pmatrix} = \frac{e^2}{h} \begin{pmatrix} 4/3 & -1 & 0 \\ -1/3 & 4/3 & -1 \\ 0 & -1/3 & 4/3 \end{pmatrix} \begin{pmatrix} V_1 \\ V_2 \\ V_3 \end{pmatrix} . \tag{S12}$$

The current is injected at contact 1 also in this configuration, so that

$$\begin{pmatrix} I_1 \\ I_2 \\ I_3 \end{pmatrix} = \begin{pmatrix} I \\ 0 \\ 0 \end{pmatrix} . \tag{S13}$$

Hence, the voltages are

$$\begin{pmatrix} V_1 \\ V_2 \\ V_3 \end{pmatrix} = I \frac{h}{e^2} \begin{pmatrix} 0.975 \\ 0.300 \\ 0.075 \end{pmatrix} , \tag{S14}$$

so that the voltages measured at contact 1 will be

$$V_1 = 0.975 \times I \frac{h}{e^2} . \tag{S15}$$

Thus, the two-terminal resistance between the contacts 1 and 4

$$R_{2-\mathrm{probe},\,14} = \frac{V_1}{I} = 0.975 \times \frac{h}{e^2} = 25.17\,\mathrm{k}\Omega . \tag{S16}$$

At the same time, the experimentally measured value of the resistance between the contacts 1 and 4 is $\approx 38.6\,\mathrm{k\Omega}$.

Thus, experimentally measured values of resistances in both configurations are very different from those that one would have in the absence of charge equilibration between counter-propagating modes in the 2/3 edge. At the same time, these values correspond exactly to the limit of strong charge equilibration between the modes. Indeed, in this limit, the edge can be thought of (in the sense of charge transport) as a single mode with the conductance $(2/3)e^2/h$, which yields the resistance $(3/2)h/e^2$ for both configurations—which is exactly the measured values. This confirms that charge equilibration is fully developed for both propagation lengths in our device.

S12. BIAS RESPONSE OF $\nu = 1/3$ STATE

In Fig.S13 we present the bias response of the $\nu = 1/3$ state in the device-1. The corresponding measurement scheme is shown in panel (a): a 100pA AC signal from the lock-in, superimposed on a DC bias current, is injected at contact C, and the AC voltage at contact C and contact B is measured. The voltage at contact C (shown in red) and contact B (shown in blue) are shown in panel (b). The voltage at contact C remains almost flat, which confirms that the conductance of the 1/3 state barely changes with applied bias. The nearly zero voltage signal at contact B demonstrates that current flows almost entirely in the downstream direction. These observations confirm that contribution of the bulk transport in the $\nu = 1/3$ state is negligibly small. This is in full consistency with a very small value of the noise measured in the upstream direction at $\nu = 1/3$, see Fig. 2d of the manuscript.

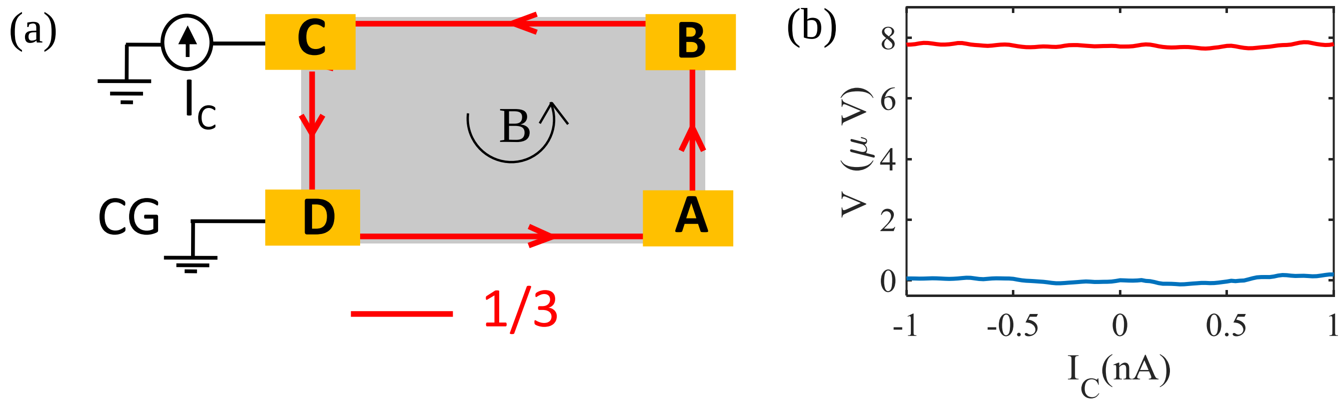

FIG. S13: (**a**) Device schematic for the bias measurement at $\nu = 1/3$ state. (**b**) The bias response at $\nu = 1/3$ state indicating negligible bulk contribution.

S13. THEORETICAL CALCULATIONS OF NOISE

A. Preliminaries

We consider a FQH edge segment with length L connected to two contacts, as depicted in Fig. S14. Our model of this segment consists of three regions: two non-interacting contact regions and one central, interacting region. We are interested in noise in the large bias regime $eV_0 \gg k_B T_0$, where e is the elementary charge, V_0 is the bias voltage, k_B is the Boltzmann constant and T_0 is the system base temperature. Under this condition, we set $T_0 = 0$ in the following. We assume the regime of strong charge equilibration along the edge segment, i.e., $L \gg l_{\rm eq}^C$, where $l_{\rm eq}^C$ is the charge equilibration length. Then, the dc noise S generated due to inter-mode tunneling along this this segment can be written as [S6–S9]

$$S = \frac{2e^2}{hl_{\rm eq}^C}\frac{\nu_-}{\nu_+}(\nu_+ - \nu_-)\int_0^L dx\ \Lambda(x)e^{-\frac{2x}{l_{\rm eq}^C}}. \tag{S17}$$

Here, h is the Planck constant, and ν_+ and ν_- are the total filling factors of the downstream and upstream edge modes, respectively, with the bulk filling factor $\nu = \nu_+ - \nu_-$. The exponential factor in the integral is a result of chiral charge transport, $\nu_+ \neq \nu_-$, and implies that the dominant noise contribution comes from a region of size $\sim l_{\rm eq}^C$ close to the left contact, the so-called noise spot. In Eq. (S17), we have neglected thermal fluctuations emanating from the contacts, as these fluctuations are much weaker than the non-equilibrium fluctuations induced by the bias, in view of the condition $eV_0 \gg k_B T_0$.

The main quantity to compute in Eq. (S17) is the local noise kernel

$$\Lambda(x) = \frac{S_{\rm loc}[\Delta V(x), T_+(x), T_-(x)]}{2g_{\rm loc}[\Delta V(x), T_+(x), T_-(x)]}, \tag{S18}$$

where $S_{\rm loc}$ and $g_{\rm loc}$ are the local electron-tunneling dc noise and the tunneling conductance, respectively. These quantities depend on microscopic details of the edge, including the inter-mode interactions, the local voltage difference between the modes $\Delta V(x)$, and the effective temperatures $T_\pm(x)$ of downstream (+) and upstream (−) edge modes. Below we compute $\Lambda(x)$, and the resulting noise S, for the edges at fillings $\nu = 2/3$ (in Sec. S13 B) and $\nu = 3/5$ (in Sec. S13 C).

Note that the previous works [S6–S9] where the theory of noise on a FQH edge was developed assumed that charge (subscript "C") and heat (subscript "H") equilibration lengths are of the same order, $l_{\rm eq}^H \sim l_{\rm eq}^C$. The focus there was on the regime of strong charge and heat equilibration, $L \gg l_{\rm eq}^H \sim l_{\rm eq}^C$.On the other hand, experimental results of Ref. [S10] (where the thermal conductance was studied) and of the present work on graphene samples show that the system is in the regime $l_{\rm eq}^C \ll L \ll l_{\rm eq}^H$ and thus the two equilibration length differ very strongly. Emergence of this regime was explained theoretically in Ref. [S10] as the effect of a strong inter-mode interaction. Specifically, it was shown there that, when a parameter Δ characterizing the interaction strength (see below for more detail) approaches unity, the

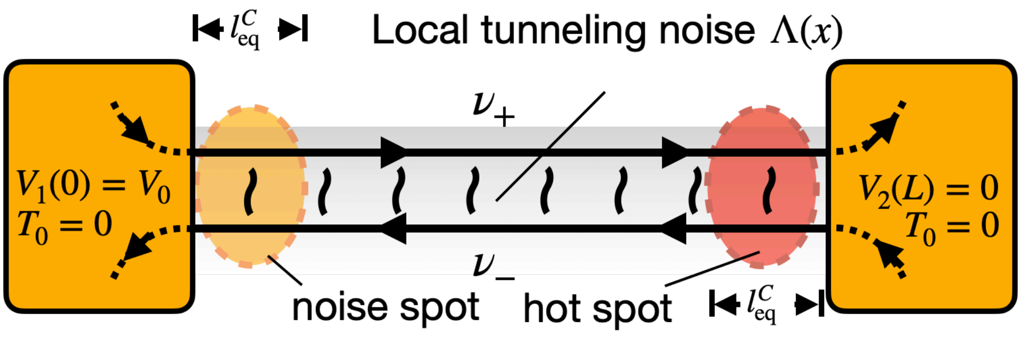

FIG. S14: Noise generation on the edge. The two contacts are biased with the voltage V_0 satisfying $eV_0 \gg k_B T_0$. The voltage drop and Joule heating occurs in a region of size $\sim l_{\rm eq}^C$ close to the right contact. This is called the hot spot. Excess dc noise is predominantly generated instead in the region of the spatial extent $\sim l_{\rm eq}^C$ close to the left contact. This is the noise spot. The generated noise is described by Eq. (S17). In the experiment, the generated noise is found by measuring the voltage fluctuations of the left contact (which is on the upstream side of the segment) that is floating and serves as a voltage probe. Left and right contacts of this figure correspond to contacts B and C in Fig. 2a of the main manuscript, respectively.

thermal equilibration diverges as $l_{\rm eq}^{H} \sim (\Delta-1)^{-1}$, while $l_{\rm eq}^{C}$ does not show this singularity. Very recently, the regime $l_{\rm eq}^{C} \ll L \ll l_{\rm eq}^{H}$ was also observed in GaAs structures [S11]. Our main goal here is the calculation of the noise (S17) for $\nu = 2/3$ and $\nu = 3/5$ edges in this regime of strong charge equilibration but vanishing thermal equilibration. Parallel to this work, a related calculation of the so-called ΔT-noise (i.e., induced by a temperature difference between the contacts rather than by applied voltage as here) was carried out in Ref. [S11].

B. Noise on the $\nu = 2/3$ edge

We consider the $\nu = 2/3$ edge hosting two counter-propagating bare modes, the downstream $\nu_+ = 1$ mode and the upstream $\nu_- = 1/3$ mode. The expectation values of the local noise $S_{\rm loc}$ and the electron tunneling conductance $g_{\rm loc}$ can be computed within the chiral Luttinger liquid model [S12, S13]. To leading order in the local tunneling strength Γ_0, these quantities are given as [S10, S14]

$$S_{\rm loc}(x) = 4\int_{-\infty}^{\infty} d\tau \cos\left[\frac{e\Delta V(x)\tau}{\hbar}\right] \langle \mathcal{T}(\tau,0)\mathcal{T}^\dagger(0,0)\rangle, \tag{S19}$$

$$g_{\rm loc}(x) = 2i\int_{-\infty}^{\infty} d\tau \tau \langle \mathcal{T}(\tau,0)\mathcal{T}^\dagger(0,0)\rangle. \tag{S20}$$

Here, the electron tunneling operator

$$\mathcal{T}(\tau,0) = \frac{\Gamma_0}{2\pi b}\exp\left[i\sqrt{\Delta-1}\phi_+(\tau,0) + i\sqrt{\Delta+1}\phi_-(\tau,0)\right], \tag{S21}$$

is written in terms of the two bosonic eigenmodes $\phi_\pm$ in the interacting region [S10], and b is a short distance cutoff. The parameter Δ quantifies the strength of interactions, with $\Delta = 2$ for vanishing interactions, and $\Delta = 1$ for strong interactions [S15, S16]. Assuming that each of the eigenmodes is in local equilibrium, with respective temperatures T_+ and T_-, the correlation functions are evaluated as

$$\langle \mathcal{T}(\tau,0)\mathcal{T}^\dagger(0,0)\rangle = \frac{|\Gamma_0|^2}{(2\pi b)^2} G_+(\tau,0)^{2d_+} G_-(\tau,0)^{2d_-}, \tag{S22}$$

with the finite temperature Green's functions

$$G_\pm(\tau,0) = \frac{\pi b T_\pm/v_\pm}{\sin\left[\frac{\pi T_\pm}{v_{L,R}}(b - i\tau v_\pm)\right]}, \tag{S23}$$

and the exponents

$$2d_+ = \Delta - 1, \tag{S24}$$

$$2d_- = \Delta + 1. \tag{S25}$$

The Fourier transforms of the factors $G_\pm(\tau,0)^{2d_\pm}$ read

$$\begin{aligned} P_\pm(\omega,T_\pm) &\equiv \int_{-\infty}^{\infty} d\tau e^{i\omega\tau} G_\pm(\tau,0)^{2d_\pm} \\ &= \left(\frac{2\pi b T_\pm}{v_\pm}\right)^{2d_\pm-1}\left(\frac{b}{v_\pm}\right) e^{\omega/(2T_\pm)} \frac{|\Gamma(d_\pm + i\frac{\omega}{2\pi T_\pm})|^2}{\Gamma(2d_\pm)}, \end{aligned} \tag{S26}$$

with $\Gamma(z)$ being the gamma-function. We also give the zero temperature limits of Eqs. (S23) and (S26), in which

$$G_\pm(\tau,0) = \frac{b}{(b - i\tau v_\pm)}, \tag{S27}$$

$$P_\pm(\omega,0) = \frac{2\pi(b/v_\pm)^{2d_\pm}\omega^{2d_\pm-1}\Theta(\omega)}{\Gamma(2d_\pm)}, \tag{S28}$$

with $\Theta(\omega)$ being the step function. With the Fourier transforms, the noise (S19) and the tunneling conductance (S20) can be expressed as

$$S_{\rm loc}(x) = \frac{4|\Gamma_0|^2}{2(2\pi b)^2}\int_{-\infty}^{\infty}\frac{d\omega}{2\pi}P_+(-\omega,T_+)\left[P_-(\omega+\frac{e\Delta V}{\hbar},T_-)+P_-(\omega-\frac{e\Delta V}{\hbar},T_-)\right], \tag{S29}$$

$$g_{\rm loc}(x) = \frac{2|\Gamma_0|^2}{(2\pi b)^2}\frac{\partial}{\partial\omega'}\left(\int_{-\infty}^{\infty}\frac{d\omega}{2\pi}P_+(\omega'-\omega,T_+)P_-(\omega,T_-)\right)\Big|_{\omega'=0}. \tag{S30}$$

For notational ease, we have here suppressed the x-dependence on ΔV and $T_\pm$. Combining Eqs. (S29) and (S30), we express the noise kernel (S18) as

$$\Lambda(x) = \frac{1}{2}\times\frac{\int_{-\infty}^{\infty}d\omega P_+(-\omega,T_+)\left[P_-(\omega+\frac{e\Delta V}{\hbar},T_-)+P_-(\omega-\frac{e\Delta V}{\hbar},T_-)\right]}{\frac{\partial}{\partial\omega'}\left(\int_{-\infty}^{\infty}d\omega P_+(\omega'-\omega,T_+)P_-(\omega,T_-)\right)\Big|_{\omega'=0}}. \tag{S31}$$

Let us briefly discuss the content of Eq. (S31). First, the noise kernel depends on the interaction strength (parameter Δ) through the exponents (S24)-(S25) entering the Fourier transforms $P_\pm$ in Eq. (S26). The noise kernel has also generally a spatial dependence via the local temperatures $T_\pm(x)$ and the local voltage difference $\Delta V(x)$. We turn now to the computation of these two quantities.

1. *Voltage drop and dissipated power along the edge*

First, we compute voltage profiles along the $\nu = 2/3$ edge to extract the voltage drop $\Delta V(x)$. Charge transport along the edge segment is described by the transport equation [S10, S17]

$$\partial_x\begin{pmatrix}I_1(x)\\ I_2(x)\end{pmatrix} = \frac{1}{l^C_{\rm eq}}\begin{pmatrix}-1 & 3\\ -1 & 3\end{pmatrix}\begin{pmatrix}I_1(x)\\ I_2(x)\end{pmatrix}, \tag{S32}$$

expressed in terms of the local bare-mode charge currents $I_{1,2}(x)$ and the charge equilibration length $l^C_{\rm eq}$. This equation should be supplemented by boundary conditions at the contacts. The natural physical picture is that the metallic contacts screen the interaction, and one has bare modes coupled to the contact [S16]. The corresponding voltages are thus applied to the bare modes emanating form the contact, yielding the boundary conditions $I_1(0) = e^2/h\times V_0$ and $I_2(L) = 0$ (see Ref. S17 for a recent detailed discussion regarding such boundary conditions). Solving Eq. (S32) and using the relations $I_1(x) = e^2V_1(x)/h$ and $I_2(x) = e^2V_2(x)/3h$, we find the local voltage drop

$$\Delta V(x) \equiv [V_1(x)-V_2(x)] = V_0\left[\frac{2}{3}\frac{e^{2x/l^C_{eq}}}{e^{2L/l^C_{eq}}-1/3}\right]. \tag{S33}$$

Equation (S33) reveals that in the limit $L \gg l^C_{eq}$, the voltage drop occurs only in a region of size $\sim l^C_{eq}$ close to the right contact (see Fig. S14), $L - x \lesssim l^C_{eq}$. We call this the hot spot since this is where the Joule heating takes place. In the rest of the segment, $\Delta V(x)$ is exponentially suppressed. In particular, at the noise spot ($x \lesssim l^C_{eq}$), the voltage drop is exponentially small [$\sim \exp(-2L/l^C_{eq})$] and can be safely approximated by zero.

We also compute the power P dissipated in the hot spot. Under the assumption of strong charge equilibration, electrical energy conservation in the edge modes leads to the formula [S8]

$$P = \frac{e^2V_0^2}{2h}\times\frac{(\nu_+-\nu_-)\nu_-}{\nu_+}. \tag{S34}$$

Applied to $\nu = 2/3$, where $\nu_+ = 1$ and $\nu_- = 1/3$, Eq. (S34) gives

$$P_{2/3} = \frac{e^2V_0^2}{9h}. \tag{S35}$$

This power becomes distributed among the edge channels and then propagates towards both contacts limiting the edge segments. (We assume no dissipation to the environment.) The resulting profile of effective temperatures $T_\pm(x)$ depend on the degree of thermal equilibration within the edge. Below we compute $T_\pm$ in the regime of negligibly weak thermal equilibration.

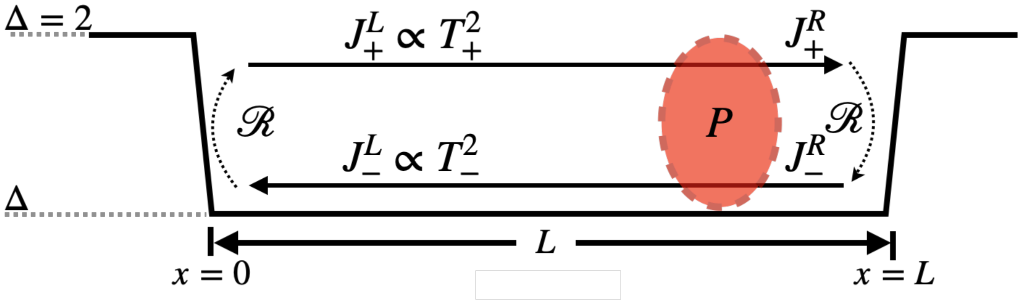

FIG. S15: Determination of steady-state temperatures $T_\pm$ on the edge (to the left of the hot spot). The dissipated power (Joule heat) P in the hot spot is distributed among left- and right-propagating eigenmodes. At interfaces with contact regions (near the points $x = 0$ and $x = L$), the heat is partially reflected (reflection coefficient $\mathcal{R}$) due to different strength of interaction in the central region (strong interaction, parameter Δ) and in contact regions (no interaction, $\Delta = 2$). This reduces the energy escape from the edge into the contacts, thus enhancing the the temperatures $T_\pm$. The steady state is determined by the system of equations (S36), which yield the temperatures (S38) and (S39).

2. *Effective temperatures*

We compute now the effective temperatures $T_\pm$. Assuming vanishing thermal equilibration, thermal transport on the edge can be modelled as in Fig. S15. The dissipated power $P_{2/3}$ [see Eq. (S35)] acts as a local heat source for the edge heat currents. To find the temperatures of the edge modes, we consider four heat currents $J_\pm^{L,R} = 0.5 \times \kappa_0 (T_\pm^{L,R})^2$ [with $\kappa_0 = \pi^2 k_B^2/(3h)$], which are right (+) and left (−) moving currents to the left (L) and to the right (R) of the heat source as depicted in Fig. S15. Power conservation then leads to the following set of equations:

$$J_+^L = \mathcal{R} J_-^L, \tag{S36a}$$
$$J_-^R = \mathcal{R} J_+^R, \tag{S36b}$$
$$J_+^R - J_+^L = P_+, \tag{S36c}$$
$$J_-^L - J_-^R = P_-. \tag{S36d}$$

Here, the first two equations describe the reflection of the heat currents due to the change in interaction strength at the contacts. Specifically, away from the contacts, the interaction is strong and characterized by the parameter Δ, while in the contact region we have no interaction, which corresponds to $\Delta = 2$. This reflection is parametrized by the reflection coefficient $\mathcal{R}$, given in terms of the interaction parameter Δ via the relations [S16]

$$\mathcal{R} = \left(\frac{1 - \sqrt{1 - c^2}}{c} \right)^2, \quad \Delta = \frac{2 - \sqrt{3}c}{\sqrt{1 - c^2}}. \tag{S37}$$

Here c is proportional to the strength of interaction and satisfies (assuming a repulsive interaction) $0 \leq c < 1$. For the strong-interaction fixed point $\Delta = 1$, we have $c = \sqrt{3}/2$, and $\mathcal{R} = 1/3$. (For vanishing interactions, $c = 0$, one has $\Delta = 2$ and $\mathcal{R} = 0$: there is no reflections when the interaction vanishes both in the contacts and in the central region.)

Since the $\nu = 2/3$ edge hosts one bosonic mode in each direction, we assume that the dissipated power is distributed equally for right and left moving currents, i.e., $P_+ = P_- = 0.5 \times P_{2/3}$ with $P_{2/3}$ given in Eq. (S35). Then, by solving Eq. (S36), we find the effective eigenmode temperatures to the left of the heat source:

$$k_B T_+ \equiv k_B T_+^L = \frac{eV_0}{\pi\sqrt{3}} \left(\frac{\mathcal{R}}{1 - \mathcal{R}} \right)^{1/2}, \tag{S38}$$

$$k_B T_- \equiv k_B T_-^L = \frac{eV_0}{\pi\sqrt{3}} \left(\frac{1}{1 - \mathcal{R}} \right)^{1/2}. \tag{S39}$$

At the strongly interacting fixed point ($\Delta = 1$), we have $\mathcal{R} = 1/3$, which yields

$$k_B T_+ = 0.13 eV_0 , \qquad k_B T_- = 0.23 eV_0 . \tag{S40}$$

Equations (S38)-(S39) indicate that reflections at the contact interfaces tend to increase the overall temperature on the edge. (For example, in the limit of absent inter-mode interaction, when $\mathcal{R} = 0$, we would have $k_B T_+ = 0$ and $k_B T_- = 0.18 eV_0$.) This is quite transparent physically, since the reflections reduce the escape of the heat from the edge into the contacts. Note that the temperatures T_+, T_- given by Eqs. (S38), (S39), (S40) are independent on the coordinate x (within the segment of the edge from the left contact to the hot spot), which is a manifestation of the absence of thermal equilibration in the edge. These formulas thus give, in particular, the effective temperatures at the noise spot, which are needed to calculate the noise as given by Eq. (S17). We proceed now with this calculation.

3. Results

We now have all the ingredients that are needed to compute the noise (S17) for the $\nu = 2/3$ edge. For the voltage drop, Eq. (S33) yields yields an exponentially small ΔV at the noise spot, and we can thus safely set it to zero. Further, we use Eqs. (S38)-(S39) for the temperatures of both eigenmodes. Since $\Lambda(x)$ in Eq. (S31) is independent of x in the considered regime of vanishing thermal equilibration, we can trivially perform the x-integration in Eq. (S17). We then arrive at

$$S_{2/3} = \frac{2e^2}{9h} \Lambda(V_0, \Delta)\,, \tag{S41}$$

where we have emphasized the dependence of Λ on the interaction parameter Δ and on the bias voltage V_0. The interaction Δ enters via the exponents (S24)–(S25) and via the reflection coefficent (S37). The voltage bias V_0 enters via the local temperatures (S38)–(S39).

We express now the noise $S_{2/3}$ in terms of the bias current

$$I_0 = \frac{2e^2}{3h} V_0\,. \tag{S42}$$

Further, we take the value $\Delta = 1$ corresponding to the strong-interaction fixed point. Experimental observation of the hierarchy of equilibration lengths, $l^C_{\rm eq} \ll l^H_{\rm eq}$, indicates that Δ is close to unity as discussed above. Equation (S41), in combination with Eq. (S31) for Λ, then yields

$$S_{2/3} = 0.146\, eI_0. \tag{S43}$$

We have checked that small deviations of Δ from the value $\Delta = 1$ lead only to very small variations of the numerical prefactor in Eq. (S43).

Equation (S43) represents our key result for the bias-induced noise on a $\nu = 2/3$ edge. Let us emphasize that the noise (S43) is independent on the length L, which is hallmark of the thermally non-equilibrated regime, $L \ll l^H_{\rm eq}$. In the opposite limit of strong thermal equilibration ($L \gg l^H_{\rm eq}$), the noise is suppressed and decays as $(L/l^H_{\rm eq})^{-1/2}$ [S6, S7]. The experimentally observed L-independence of the noise (Fig. 2d of the main manuscript) thus provides a clear evidence of the thermally non-equilibrated regime. The theoretical formula (S43) is compared with our experimental results in Fig. 2d of the main manuscript and in Fig. S11b of this Supplementary Material. The excellent agreement between the theoretical and experimental magnitude of the noise provides further strong support to our interpretation of the experiment.

C. Noise at filling 3/5

The edge structure at $\nu = 3/5$ is more complicated than at $\nu = 2/3$, since the edge of a hole-conjugated $\nu = 3/5$ state hosts three bare edge modes: ϕ_j with $j = 1, 2, 3$ and $\nu_1 = 1$, $\nu_2 = 1/3$, and $\nu_3 = 1/15$. Whereas ϕ_1 is propagating downstream, ϕ_2 and ϕ_3 are upstream modes [S18, S19].

To capture the essential physics on this edge, we apply three approximations. First, we note that charge tunneling between the two co-propagating upstream modes can not partition charges between the two contacts. Such processes therefore do not influence the noise and can be ignored. Second, we assume an efficient equilibration between the two upstream modes, so that the edge essentially consists of two effective counter-propagating, hydrodynamic modes with $\nu_+ = \nu_1 = 1$ and $\nu_- = \nu_2 + \nu_3 = 1/3 + 1/15 = 2/5$ (see Fig. S14). Third, in order to simplify the technical analysis, we assume that electron tunneling between the adjacent ϕ_1 and ϕ_2 modes is much stronger than that between ϕ_1 and ϕ_3 which are further apart. The renormalization of these two tunneling processes follow the same renormalization group equations [S19], so if the difference in tunneling amplitudes is large at some energy, it will remain large during

renormalization to lower energies. In principle, any of this approximations can be relaxed. While this would make calculations substantially more cumbersome, one would end up with nearly the same result, up to a small variation of the numerical prefactor.

With these simplifications, the microscopic description of the noise kernel $\Lambda(x)$ [see Eq. (S18)] becomes exactly the same as the one for the $\nu = 2/3$ edge, i.e., it is given by Eq. (S31). What remains to be computed for the $\nu = 3/5$ edge is then the voltage drop $\Delta V(x)$ and the effective temperatures $T_\pm$ of downstream and upstream eigenmodes in the interacting region. (Here T_- is the temperature of both upstream eigenmodes since, in order to simplify the analysis, they are assumed above to equilibrate efficiently.)

1. Voltage drop and dissipated power along the edge

The analysis of Sec. S13 B 1 is straightforwardly extended to the $\nu = 3/5$ edge. In full analogy with to the $\nu = 2/3$ edge, the voltage drop at the $\nu = 3/5$ edge occurs only close to the right contact and is exponentially suppressed away from it [S7]. In particular, the voltage drop $\Delta V(x)$ is exponentially suppressed in L/l_{eq}^C at the noise spot (near the left contact) and can be approximated by zero.

To compute the dissipated power, we apply Eq. (S34) with $\nu_+ = 1$ and $\nu_- = 2/5$. The result is

$$P_{3/5} = \frac{3e^2V_0^2}{25h}. \tag{S44}$$

We note that dissipated powers $P_{2/3}$ [see Eq. (S35)] and $P_{3/5}$ are very close in magnitude.

2. Effective temperatures

We consider four heat currents $J_\pm^{L,R} = 0.5 \times n_\pm \times \kappa_0 (T_\pm^{L,R})^2$, denoting right (+) and left (−) moving currents to the left (L) and to the right (R) of the heat source (see Fig. S15). At variance with the $\nu = 2/3$ edge, we need here to take into account the difference in the number of downstream and upstream modes. This is done by including the numbers $n_+ = 1$ and $n_- = 2$ in the heat currents above. Power conservation on the $\nu = 3/5$ edge leads to the following system of equations that represents an extension of the system (S36):

$$J_+^L = \frac{1}{2}\mathcal{R} J_-^L, \tag{S45a}$$

$$J_-^R = \mathcal{R} J_+^R, \tag{S45b}$$

$$J_+^R - J_+^L = P_+, \tag{S45c}$$

$$J_-^L - J_-^R = P_-. \tag{S45d}$$

Note the factor of 1/2 in Eq. (S45a) as compared to Eq. (S36a). This is a consequence of the difference in the number of downstream and upstream modes.

We assume that the dissipated power $P_{3/5}$ [given by Eq. (S44)] is distributed proportionally to the number of downstream and upstream modes (n_+ and n_-), so that $P_+ = (1/3)P_{3/5}$ and $P_- = (2/3)P_{3/5}$. Further, the reflection coefficient $\mathcal{R}$ depends on the interactions between the modes of the 3/5 edge. In analogy with the above analysis of the 2/3 edge, we assume that the system is close to the strong-interaction fixed point (counterpart of $\Delta = 1$ point of the 2/3 edge). As discussed above, this assumption is consistent with experimental observation of nearly vanishing thermal equilibration. At the strongly interacting fixed point, the reflection coefficient for the 3/5 edge reads [S11]

$$\mathcal{R} = 1 - \nu = 2/5. \tag{S46}$$

The solution to (S45) for the temperatures to the left of the hot spot (in particular, at the the noise spot) then becomes

$$k_B T_+ \equiv k_B T_+^L = \sqrt{\frac{2}{23}} \times \frac{6eV_0}{5\pi} \approx 0.11 eV_0, \tag{S47}$$

$$k_B T_- \equiv k_B T_-^L = \sqrt{\frac{1}{115}} \times \frac{6eV_0}{\pi} \approx 0.18 eV_0. \tag{S48}$$

Both temperatures are somewhat below their values for the 2/3 edge, Eq. (S40), but the differences are not too big. For comparison, if one assumes no interaction on the edge, and thus $\mathcal{R} = 0$, one gets the temperatures $k_B T_+ = 0$ and $k_B T_- \approx 0.16 eV_0$.

3. Digression: Temperatures in the case of a generic edge

As a short digression, we compute the effective temperatures $T_\pm$ for the case of generic $n_\pm$ by extending the analysis of Ref. S13 B 2 and S13 C 2. We consider four heat currents $J_\pm^{L,R} = 0.5 \times n_\pm \times \kappa_0 (T_\pm^{L,R})^2$ [with $\kappa_0 = \pi^2 k_B^2/(3h)$] denoting right (+) and left (−) moving currents to the left (L) and to the right (R) of the heat source. We assume that all downstream (upstream) modes have the same temperature T_+ (respectively, T_-). Power conservation then leads to the following set of equations:

$$J_+^L = \frac{\mathcal{R}}{n_-} J_-^L, \tag{S49a}$$

$$J_-^R = \frac{\mathcal{R}}{n_+} J_+^R, \tag{S49b}$$

$$J_+^R - J_+^L = P_+ = P \times \frac{n_+}{n_+ + n_-}, \tag{S49c}$$

$$J_-^L - J_-^R = P_- = P \times \frac{n_-}{n_+ + n_-}. \tag{S49d}$$

Solving for $J_\pm^L$, we obtain

$$J_+^L = P \times \frac{\mathcal{R} n_+ (n_- + \mathcal{R})}{(n_+ + n_-)(n_+ n_- - \mathcal{R}^2)}, \tag{S50}$$

$$J_-^L = P \times \frac{n_+ n_- (n_- + \mathcal{R})}{(n_+ + n_-)(n_+ n_- - \mathcal{R}^2)}. \tag{S51}$$

Converting these currents to temperatures, we find

$$k_B T_+^L = \left(\frac{6hP}{\pi^2} \times \frac{\mathcal{R}(n_- + \mathcal{R})}{(n_+ + n_-)(n_+ n_- - \mathcal{R}^2)} \right)^{1/2}, \tag{S52}$$

$$k_B T_-^L = \left(\frac{6hP}{\pi^2} \times \frac{n_+ (n_- + \mathcal{R})}{(n_+ + n_-)(n_+ n_- - \mathcal{R}^2)} \right)^{1/2}. \tag{S53}$$

For $P = P_{2/3} = e^2 V_0^2/9h$, $n_+ = 1$, $n_- = 1$, and $\mathcal{R} = 1/3$, we recover $k_B T_+^L \approx 0.13 eV_0$ and $k_B T_-^L \approx 0.23 eV_0$, Eq. (S40). For $P = P_{3/5} = 3e^2 V_0^2/25h$, $n_+ = 1$, $n_- = 2$ and $\mathcal{R} = 2/5$, we recover $k_B T_+^L \approx 0.11 eV_0$ and $k_B T_-^L \approx 0.18 eV_0$, Eqs. (S47) and (S48).

4. Results for the noise on the $\nu = 3/5$ edge

After a short digression in Sec. S13 C 3, we finalize the calculation of the noise for $\nu = 3/5$. Performing the integration over x (which is trivial due to constant temperatures in the noise spot) in Eq. (S17), we obtain for the noise on the $\nu = 3/5$ edge

$$S_{3/5} = \frac{6e^2}{25h} \Lambda(V_0, \Delta) , \tag{S54}$$

which is a counterpart of the $\nu = 2/3$ formula (S41). For the strong-interaction fixed point ($\Delta = 1$), the the temperatures are given by Eqs. (S47) and (S48). Using them in Eq. (S31), we determine Λ, which yields, by virtue of Eq. (S54), the noise $S_{3/5}$. Expressing it in terms of the bias current

$$I_0 = \frac{3e^2}{5h} V_0 , \tag{S55}$$

we finally obtain

$$S_{3/5} = 0.138 \, eI_0 . \tag{S56}$$

Note that the noise is very close to that on the 2/3 edge, Eq. (S43). The theoretical result (S56) agrees very well with our experimental data for the noise on the 3/5 edge, see Fig. S11c.

It is interesting to compare the result (S56) obtained for the thermally non-equilibrated 3/5 edge ($L \gg l^H_{\rm eq}$) to the noise $S_{3/5,{\rm eq}}$ found on the same edge in the opposite limit of strong thermal equilibration ($L \ll l^H_{\rm eq}$). The latter calculation was carried out in Ref. S7, where it was found that

$$S_{3/5,{\rm eq}} = 0.116\, eI_0. \tag{S57}$$

We see that the magnitudes of noise in the two limits differ by approximately 20%. The difference is much less dramatic than in the case of the $\nu = 2/3$ noise (which is parametrically suppressed in the thermally equilibrated regime). This is because, in the regime of strong thermal equilibration, the upstream heat transport remains ballistic on the 3/5 edge but becomes diffusive on the 2/3 edge.

SUPPLEMENTARY REFERENCES:

[S1] D. Purdie, N. Pugno, T. Taniguchi, K. Watanabe, A. Ferrari, and A. Lombardo, Nature communications **9**, 1 (2018).
[S2] F. Pizzocchero, L. Gammelgaard, B. S. Jessen, J. M. Caridad, L. Wang, J. Hone, P. Bøggild, and T. J. Booth, Nature communications **7**, 1 (2016).
[S3] Y. Kim, P. Herlinger, T. Taniguchi, K. Watanabe, and J. H. Smet, ACS nano **13**, 14182 (2019).
[S4] D. B. Gutman, I. V. Protopopov, A. L. Burin, I. V. Gornyi, R. A. Santos, and A. D. Mirlin, Phys. Rev. B **93**, 245427 (2016).
[S5] M. Büttiker, Physical Review B **38**, 9375 (1988).
[S6] J. Park, A. D. Mirlin, B. Rosenow, and Y. Gefen, Phys. Rev. B **99**, 161302 (2019), ISSN 24699969.
[S7] C. Spånslätt, J. Park, Y. Gefen, and A. D. Mirlin, Phys. Rev. Lett. **123**, 137701 (2019).
[S8] C. Spånslätt, J. Park, Y. Gefen, and A. D. Mirlin, Phys. Rev. B **101**, 075308 (2020).
[S9] J. Park, C. Spånslätt, Y. Gefen, and A. D. Mirlin, Phys. Rev. Lett. **125**, 157702 (2020).
[S10] S. K. Srivastav, R. Kumar, C. Spånslätt, K. Watanabe, T. Taniguchi, A. D. Mirlin, Y. Gefen, and A. Das, Phys. Rev. Lett. **126**, 216803 (2021).
[S11] R. A. Melcer, B. Dutta, C. Spånslätt, J. Park, A. D. Mirlin, and V. Umansky, arXiv preprint arXiv:2106.12486 (2021).
[S12] X. G. Wen, Phys. Rev. B **41**, 12838 (1990).
[S13] A. M. Chang, Rev. Mod. Phys. **75**, 1449 (2003).
[S14] J. Rech, T. Jonckheere, B. Grémaud, and T. Martin, Phys. Rev. Lett. **125**, 086801 (2020).
[S15] C. L. Kane, M. P. A. Fisher, and J. Polchinski, Phys. Rev. Lett. **72**, 4129 (1994).
[S16] I. Protopopov, Y. Gefen, and A. Mirlin, Annals of Physics **385**, 287 (2017), ISSN 0003-4916.
[S17] C. Spånslätt, Y. Gefen, I.V. Gornyi, and D.G. Polyakov, arXiv preprint arXiv:2105.04013 (2021).
[S18] C. L. Kane and M. P. A. Fisher, Phys. Rev. B **51**, 13449 (1995).
[S19] J. E. Moore and X.-G. Wen, Phys. Rev. B **57**, 10138 (1998).
